# Visually assessed breast density, breast cancer risk and the importance of the craniocaudal view

**DOI:** 10.1186/bcr2123

**Published:** 2008-07-23

**Authors:** Stephen W Duffy, Iris D Nagtegaal, Susan M Astley, Maureen GC Gillan, Magnus A McGee, Caroline RM Boggis, Mary Wilson, Ursula M Beetles, Miriam A Griffiths, Anil K Jain, Jill Johnson, Rita Roberts, Heather Deans, Karen A Duncan, Geeta Iyengar, Pam M Griffiths, Jane Warwick, Jack Cuzick, Fiona J Gilbert

**Affiliations:** 1 Department of Epidemiology, Mathematics and Statistics, Wolfson Institute of Preventive Medicine, Charterhouse Square, London EC1M 6BQ, UK; 2 Department of Pathology, University Medical Center St Radboud, Geert Grooteplein 9, 6500 HB Nijmegen, the Netherlands; 3 Department of Imaging Science and Biomedical Engineering, University of Manchester, Oxford Road, Manchester M13 9PT, UK; 4 Department of Radiology, University of Aberdeen, Foresterhill Aberdeen AB25 2ZD, UK; 5 Department of Public Health and General Practice, Christchurch School of Medicine & Health Sciences, Riccarton Avenue, Christchurch 8140, New Zealand; 6 Nightingale Centre, Withington Hospital, Southmoor Road, Manchester M23 9LT, UK; 7 Northeast Scotland Screening Centre, Forester Hill Road, Aberdeen AB25 2XF, UK

## Abstract

**Introduction:**

Mammographic density is known to be a strong risk factor for breast cancer. A particularly strong association with risk has been observed when density is measured using interactive threshold software. This, however, is a labour-intensive process for large-scale studies.

**Methods:**

Our aim was to determine the performance of visually assessed percent breast density as an indicator of breast cancer risk. We compared the effect on risk of density as measured with the mediolateral oblique view only versus that estimated as the average density from the mediolateral oblique view and the craniocaudal view. Density was assessed using a visual analogue scale in 10,048 screening mammograms, including 311 breast cancer cases diagnosed at that screening episode or within the following 6 years.

**Results:**

Where only the mediolateral oblique view was available, there was a modest effect of breast density on risk with an odds ratio for the 76% to 100% density relative to 0% to 25% of 1.51 (95% confidence interval 0.71 to 3.18). When two views were available, there was a considerably stronger association, with the corresponding odds ratio being 6.77 (95% confidence interval 2.75 to 16.67).

**Conclusion:**

This indicates that a substantial amount of information on risk from percentage breast density is contained in the second view. It also suggests that visually assessed breast density has predictive potential for breast cancer risk comparable to that of density measured using the interactive threshold software when two views are available. This observation needs to be confirmed by studies applying the different measurement methods to the same individuals.

## Introduction

Mammographic breast density is a well established risk factor for breast cancer [1-3]. It is of particular interest because it has a high attributable fraction and because it is amenable to change in response to hormonal therapy, weight change, or diet [2,4,5]. Density is a reflection of the amount of fibroglandular, as opposed to fatty-replaced, tissue in the breast. There is evidence that breast density measured quantitatively, either as total dense area or percentage of dense area on the mammogram, is a better predictor of breast cancer risk than density classified into quantitative categories [3]. The Cumulus interactive threshold software is widely considered to be the 'gold standard' tool for density measurement [1]. With Cumulus, the reader identifies the boundaries of the breast tissue and the threshold for dense tissue on the mammogram, and the computer software calculates the total area of the breast, the total area of dense tissue and the percentage area of dense tissue.

The absolute and percentage areas of breast tissue represent a reduction of the three-dimensional volume of fibroglandular tissue in the breast to a two-dimensional projection. Intuitively, a direct measure of the volume of dense tissue is desirable. Volumetric methods are available [6-8], although these have not been studied as extensively as area-based methods [9]. Density, as measured using

Cumulus, currently has the strongest published relationship with breast cancer risk [1,3]. Percent breast density is an estimate of the proportion of fibroglandular tissue as opposed to fat.

Estimating density for large numbers of mammograms using the Cumulus software requires a major commitment of human resources. For this reason, some large studies still use visually assessed breast density [3,5]. Here, we report on density assessed using a visual analogue scale in a study of 10,048 mammograms, including those of 311 breast cancer patients in two high-throughput breast screening centres. We estimate the effect of visually measured percent density on breast cancer risk and how this is modified by differences between centres and by other variables.

## Materials and methods

Density was measured in the context of the Computer Aided Detection Evaluation Trial 1 (CADET1) [10]. In this study 10,096 mammograms, including those of 315 breast cancer cases diagnosed contemporaneously or within the following 6 years, previously double read in the UK National Breast Screening Programme in 1996, were re-read by a single reader assisted by the R2 ImageChecker computer aided detection system [11]. Cancers were identified from the screening unit records, augmented by the local cancer registries. In addition to attempting to identify cancers, the single reader also visually estimated the percent mammographic density. A straight line was provided on the proforma with its right hand end corresponding to 100% density and its left hand end to 0%. The reader examined the films and marked the point on the line corresponding to their estimate of the average density (of the two breasts and, where available, of the two views). All mammograms were read in batches, a session (morning or afternoon) at a time.

The radiologists were all experienced breast screening readers, attaining the UK breast screening programme standard of reading at least 5,000 screening mammograms per year. They did not receive special training in density measurement.

Data on density was available in 10,048 cases (99.5%), including 311 breast cancers diagnosed either at the original 1996 screen or subsequently. The single reader was not one of the original two readers, and was not aware of the final diagnosis or of the previous reading results at the time of scoring the density. The mean age at the original screen was 57 years (standard deviation [SD] = 4.8 years) The age range was from 50 to 84 years, with 96% being aged 50 to 64 years (in the UK programme at the time, women aged 50 to 64 years were regularly invited but older women were entitled to be screened on request).

Local research ethics committee approval was obtained, informed consent was not required and all mammograms were anonymized.

The study took place in two screening centres, Aberdeen and Manchester. In each centre, the re-reading duties were shared between four radiologists. Density was coded as a percentage of the bright, opaque area on the breast image. The association of density with risk was assessed using logistic regression [12]. Variation between centres and readers was analyzed by subgroup analyses using logistic regression and by considering mean and SD of density by reader and case status (cancer/noncancer). We estimated the odds ratios (ORs) associated with percent density both as a continuous variable and classified into the six categories described by Boyd and coworkers [2] (0%, 1% to 10%, 11% to 25%, 26% to 50%, 51% to 75%, and 76% to 100% dense), but combining the two lowest categories because none of the cancer patients had 0% density. In addition, we investigated potential confounders of density, including age of screenee, size, node status and grade of the cancers, and number of mammographic views. The population attributable fraction was calculated using the formula quoted by Breslow and Day [13].

It should be noted that this research was not intended to assess the correlation between visually measured and machine-measured density. The main object was to quantify the association of visually determined density with risk, and identify variables that modify or qualify the association.

## Results

Table 1 shows numbers of subjects, and mean and SD density by detection mode. The highest average density on the screening radiograph was observed for interval cancers occurring within 1 year of the 1996 screen.

**Table 1:** Number of subjects and mean density by diagnostic category

Diagnostic category	Number of subjects	Mean (SD) percent density
No cancer	9,737	33.1 (20.6)
Cancer at 1996 screen	82	42.9 (17.8)
Interval cancer < 1 year	7	50.3 (28.1)
Interval cancer 2 to 3 years	42	34.2 (22.0)
Cancer at subsequent screen	96	34.5 (21.2)
Cancer thereafter	84	42.4 (24.3)

Total	10,048	33.3 (20.7)

SD, standard deviation.

Table 2 shows the numbers of cancers and noncancers classified into five density categories, and the corresponding logistic regression OR estimates and 95% confidence intervals (CIs) adjusted for age. The results are shown for all subjects and then for each centre separately. The overall results suggest a significant effect of density on breast cancer risk, with a threefold risk for density greater than 75% compared with 10% or less. This, however, conceals a highly significant heterogeneity of the effect between centres (*P *< 0.001). The effect of density on risk was very strong in Manchester and weak in Aberdeen.

**Table 2:** Effect of categorized density on breast cancer risk by centre, age-adjusted

Population	Percent breast density (%)	Number (%) of cancers	Number (%) of noncancers	OR (95% CI)	Significance (trend)
All subjects	0 to 10	18 (6)	1022 (11)	1.00 (-)	*P *< 0.001
	11 to 25	76 (24)	2997 (31)	1.43 (0.85 to 2.42)	
	26 to 50	117 (38)	3519 (36)	1.87 (1.13 to 3.11)	
	51 to 75	82 (26)	1875 (19)	2.44 (1.45 to 4.10)	
	76 to 100	18 (6)	324 (3)	3.07 (1.57 to 5.99)	

Manchester	0 to 10	2 (1)	367 (7)	1.00 (-)	*P *< 0.001
	11 to 25	24 (18)	1397 (28)	3.14 (0.73 to 13.35)	
	26 to 50	60 (44)	2229 (44)	4.89 (1.18 to 20.08)	
	51 to 75	41 (30)	951 (19)	7.66 (1.83 to 31.93)	
	76 to 100	8 (6)	103 (2)	13.53 (2.81 to 65.10)	

Aberdeen	0 to 10	16 (9)	655 (14)	1.00 (-)	*P *= 0.12
	11 to 25	52 (30)	1600 (34)	1.33 (0.75 to 2.35)	
	26 to 50	57 (32)	1290 (28)	1.80 (1.02 to 3.17)	
	51 to 75	41 (23)	924 (20)	1.80 (0.99 to 3.25)	
	76 to 100	10 (6)	221 (5)	1.82 (0.81 to 4.10)	

CI, confidence interval; OR, odds ratio.

The Aberdeen screenees were on average 3 months older than the Manchester women. However, neither age of screenee nor tumour features accounted for the heterogeneity between centres (data available from the authors). Number of views was, however, associated with the predictive potential of density. Table 3 shows the age adjusted effect of density on risk as estimated from one view and from two views. Two views were available in 2,912 cases (29%) and one view in 7,090 (71%). For 46 cases (< 0.5%), the number of views available was not recorded. Thus, analyses incorporating number of views are based on 10,002 subjects rather than 10,048. Where only the mediolateral oblique (MLO) view was available, there was a modest, borderline significant (*P *= 0.05) effect of breast density on risk, with an OR for the highest category relative to the lowest of 1.88 (95% CI = 0.80 to 4.45). When two views were available, there was a considerably stronger association (*P *< 0.001), with the corresponding OR being 16.89 (95% CI = 2.13 to 133.87). The heterogeneity between one view and two views of the association with risk was significant (*P *= 0.002). The very wide 95% CIs in those with two views are due to the fact that the reference category of density, 0% to 10%, contained only one cancer. The difference in results remained strong when we merged the two lowest density categories to include 0% to 25% density. The OR for 76% to 100% density relative to 0% to 25% was 1.51 (95% CI = 0.71 to 3.18) when only the MLO view was available, but it was 6.77 (95% CI = 2.75 to 16.67) when two views were available.

**Table 3:** Association of categorised density with risk by number of views available

Percent breast density (%)	Single view		Two views	
	
	Number of cancers/subjects	OR (95% CI)	Number of cancers/subjects	OR (95% CI)
0 to 10	17/794	1.00 (-)	1/241	1.00 (-)
11 to 25	67/2,322	1.33 (0.77 to 2.29)	9/736	2.99 (0.37 to 23.70)
26 to 50	91/2,567	1.64 (0.96 to 2.77)	26/1,052	6.16 (0.83 to 45.63)
51 to 75	54/1,222	1.97 (1.12 to 3.43)	28/727	9.79 (1.32 to 72.47)
76 to 100	8/185	1.88 (0.79 to 4.45)	10/156	16.89 (2.13 to 133.87)
Significance	-	*P *= 0.05	-	*P *< 0.001

CI, confidence interval; OR, odds ratio.

Table 4 shows the distribution of cancers by detection mode, and noncancers, for one-view and two-view cases separately.

**Table 4:** Number of views by detection mode

Detection mode	One view (*n *[%])	Two view (*n *[%])
Noncancer	6,853	2,838
Prevalence screen	1	19
Interval cancer	101	32
Incidence screen	135	23

Total	7,090	7,090

Table 5 shows the mean and SD of density by centre, number of views and cancer status, and the age-adjusted ORs associated with an increase of 10% in density. Population attributable fractions, for a reference level of 20% density, are also shown. In Aberdeen the density determined using two views (MLO and craniocaudal [CC]) was associated with risk of breast cancer, whereas the one view (MLO only) was not. In addition, where two views were available, the estimated average densities of the cancers and noncancers exhibited good agreement between the two centres. The heterogeneity between centres with respect to the density/risk association was no longer significant when analysis was restricted to the two-view mammograms (*P *= 0.4). The percentage of cases estimated to be attributable to density in Aberdeen was much closer to that of Manchester in the case of two views.

**Table 5:** Association of continuous density with risk, by centre and number of views available

Centre	Number of views	Number of subjects	Mean (SD) density, cancers	Mean (SD) density, noncancers	OR per 10% density (95% CI)	% attributable fraction to densities > 20%
Manchester	1	3,416	39.8 (18.5)	32.2 (17.5)	1.22 (1.09 to 1.36)	3
	2	1,721	51.1 (19.7)	37.4 (19.7)	1.41 (1.19 to 1.66)	35

Aberdeen	1	3,674	32.2 (21.1)	30.6 (21.8)	1.03 (0.95 to 1.11)	30
	2	1,191	52.0 (24.0)	36.8 (24.4)	1.28 (1.11 to 1.46)	50

CI, confidence interval; OR, odds ratio; SD, standard deviation.

After exclusion of the 82 cancers detected at the evaluation screen and the seven interval cancers diagnosed within 1 year of the screen, the results were largely unchanged. The modifying effect of number of views was still strong. For one-view mammograms, the OR per 10% increase in density was 1.05 (95% CI = 0.98 to 1.14; *P *= 0.2). With two views, the OR was 1.33 (95% CI = 1.17 to 1.52; *P *< 0.001). Table 6 shows the mutually adjusted effects of density, reader within centre and age on risk, also excluding the evaluation screen cancers and the interval cancers within 1 year. The effect of density is very similar to the age-adjusted effect shown in Table 2 for all subjects and cancers.

**Table 6:** Multivariate model of breast cancer risk including density, reader/centre and age, excluding cancers diagnosed at or within 1 year of the evaluation screen

Risk factor	Category	OR (95% CI)
Percent density (%)	0 to 10	1.00 (-)
	11 to 25	1.26 (0.73 to 2.15)
	26 to 50	1.84 (1.06 to 3.19)
	51 to 75	2.09 (1.17 to 3.72)
	76 to 100	2.97 (1.41 to 6.26)

Reader Aberdeen	1	1.00 (-)
	2	0.78 (0.43 to 1.39)
	3	1.85 (1.10 to 3.09)
	4	2.04 (1.25 to 3.33)

Reader Manchester	5	0.68 (0.36 to 1.29)
	6	1.04 (0.61 to 1.76)
	7	0.79 (0.45 to 1.40)
	8	0.52 (0.27 to 1.00)

Age	Per year	0.98 (0.95 to 1.02)

CI, confidence interval; OR, odds ratio.

## Discussion

Our results indicate that there is a strong association of visually assessed percent breast density with breast cancer risk, but that the association is considerably more consistent when two views are available. The magnitude of the association with breast cancer risk is smaller than that observed using the interactive threshold software when only a single view is available, but is comparable with that derived using the software when assessed using two views [1,3]. In the latter case, a sixfold increase in risk was observed in the 76% to 100% category relative to the 0% to 25% category. The greater effect of density in Manchester is partly a reflection of the higher proportion with two views in that centre (34% in Manchester versus 24% in Aberdeen). The better risk prediction with two views may be a reflection of better performance of an assessed average density from the two views or it may be due to the CC view alone. At any rate, the results emphasize the importance of the CC view in visual assessment of breast density.

The readers were blind to the disease state of the screenees. The cancers did, however, include 82 cases that had been diagnosed at the screen pertinent to the mammograms read. When these were excluded the results were largely unchanged. When the interval cancers diagnosed within 12 months of the screen were also excluded, the results remained the same.

To investigate the possibility that the Manchester readers were first identifying cancers and then involuntarily marking higher densities, we estimated the association of density with risk in Manchester excluding those cancer cases identified by the reader. The results were similar to those using the entire tumour population; for a 10% increase in density there was an OR of 1.23 (95% CI = 1.09 to 1.39), excluding identified cancers; including all 311 cancers, the figure was 1.27 (95% CI = 1.16 to 1.39).

It is interesting to note in Table 5 that where two views were available, average estimated density was higher regardless of centre, and was slightly greater for cancers than for noncancers. Although we only have one percent density estimate per subject, not separate scores for the MLO and CC views, this result is consistent with the finding of Warren and coworkers [13] that density estimates on the CC view tend to be higher than on the MLO. It is possible that the MLO view includes fatty tissue immediately in front of the chest wall, which is not seen on the CC view, thus giving a lower percentage of dense tissue in the MLO. Also, our observation that the highest average density was observed in women with interval cancers arising within 1 year of a negative screen is consistent with the findings of Boyd and colleagues [1], who found that women with greater than 75% density were 17.8 times more likely than women with less than 10% density to develop breast cancer within 12 months of a negative screen. Our findings therefore suggest a genuine increase in risk, possibly artificially augmented by a masking effect in interval cancers arising soon after a screen.

It should be noted that the interactive threshold method will avoid some of the subjectivity to which visual assessment is prone. Also, the use of total dense area rather than percent density in the interactive threshold method might show greater consistency between subjects in its predictive potential. At least one study has found that the total dense area is a better predictor of risk [14], although the bulk of the published evidence favours percent density [15-19].

It is worth noting that availability of two views was highly confounded with whether the screen was a prevalent or incident screen. Of the prevalent screens 99% were two view, and only 3% of the incident screens were two view. The same percentages apply to the cancers. This is because in the National Screening Programme in 1997, the policy was to use two-view mammography at the prevalent screen and single-view thereafter. The policy has since been changed to two views as standard at all screens. This indicates that the number of views was determined by the prevalent/incident status of the screen and not by clinical or radiological considerations. Analysis restricted to two-view screens yielded a stronger effect of density on cancer risk than did analysis restricted to prevalent screens. This suggests that the number of views is the factor responsible for the performance of visually assessed density in predicting risk, rather than the order of screen. Similarly, confounding with age did not explain the heterogeneity by number of views.

Although breast density remains a highly important risk factor for breast cancer, the question of whether alteration of density, particularly percentage density, in an individual will necessarily alter that individual's future breast cancer risk remains open. Patel and coworkers [20], using both an automated volumetric method and a visually estimated percentage of glandular tissue, found that changes in weight resulted in dramatic differences to the percentage density but little difference in the absolute volume of fibroglandular tissue.

Nevertheless, it is increasingly clear that mammographic density has considerable potential in risk assessment to select high-risk populations or for early detection or prevention projects and programme policies. There is evidence that density measured by the Cumulus interactive threshold software is a better predictor of risk than density assessed visually [3]. The scanning and processing of the mammographic images and the subsequent operation of the software add up to a major commitment of resources.

It should be noted that important confounders of density such as body mass index and various hormonal breast cancer risk factors were not available. It is likely that had we been able to adjust for body mass index, the relation of density to risk would have been stronger [3]. Differences in body mass or other risk factor prevalences might also explain some of the difference in levels of density in the two centres, but they would be unlikely to explain the difference in the association with risk or the modifying effect of number of views.

## Conclusion

The results here suggest that a predictive power similar to that of Cumulus can be derived from visually assessed density if the CC view is available. There is, however, a need to study this question with independent estimation of density in the same subjects, using four methods: the Cumulus software; visually assessed density from a MLO view alone; visually assessed density from the CC view alone; and visually assessed density from a MLO plus CC views.

## Abbreviations

CC = craniocaudal; CI = confidence interval; MLO = mediolateral oblique; OR = odds ratio; SD = standard deviation.

## Competing interests

The authors declare that they have no competing interests.

## Authors' contributions

SWD contributed to the study conception and design, supervised the statistical analysis and drafted the manuscript. IDN performed the statistical analysis and assisted in drafting the manuscript. SA contributed to the study conception and design, and assisted in drafting the manuscript. MGCG was the study co-ordinator, contributed to study design and assisted in drafting the manuscript. MAM was responsible for medical informatics, managed the Aberdeen data and assisted in drafting the manuscript. CRMB led the Manchester centre, read the mammograms and assisted in drafting the manuscript. MW, UMB, MAG, AKJ, JJ, RMR, HD, KAD and GI read the mammograms and assisted in drafting the manuscript. PMG was responsible for conduct and data management of the Manchester data and assisted in the drafting of the manuscript. JW contributed to interpretation of results and drafting the manuscript. JC contributed to statistical analysis and drafting the manuscript. FJG acted as the study principal investigator, contributed to design, conception and conduct of the study, and assisted in the drafting of the manuscript.
